# Financial Hardship on Food Security in Ageing Populations

**DOI:** 10.3389/ijph.2023.1605755

**Published:** 2023-12-14

**Authors:** Sirinya Phulkerd, Sasinee Thapsuwan, Aphichat Chamratrithirong, Rossarin Soottipong Gray, Umaporn Pattaravanich, Chantana Ungchusak, Pairoj Saonuam

**Affiliations:** ^1^ Institute for Population and Social Research, Mahidol University, Salaya, Thailand; ^2^ Thai Health Promotion Foundation, Bangkok, Thailand

**Keywords:** financial hardship, socio-demographic characteristics, food security, ageing population, Thailand

## Abstract

**Objective:** This study investigated the prevalence of food security, and the association of food security with financial hardship and socio-demographic characteristics among the ageing population in Thailand.

**Methods:** The study extracted data on 1,197 persons age 60 years or older from a nationally-representative sample survey of Thai households. The food security data were collected using the Food Insecurity Experience Scale (FIES), developed by the Food and Agriculture Organization. Multiple regression analysis was used to investigate the association between financial hardship, socio-demographic characteristics, and food security.

**Results:** Of the total sample, 71% had food security. The least probability of having food security was observed in the respondents who sometimes and often had income problems (*p* < 0.001), and felt dissatisfied with their financial situation (*p* < 0.001). The respondents who were female, at oldest-old age, with lower than primary school education and in the Northeast were less likely to have food security.

**Conclusion:** These findings suggest the need for government assistance for those who are experiencing financial hardship to help them manage their finances and food security more effectively, taking into account different socio-demographic characteristics.

## Introduction

Many countries in the world are experiencing a faster pace of population ageing than in the past. In 2020, the global population age 60 years or older outnumbered the population under age 5 years [1]. It is expected that, by 2050, the older population will have almost doubled compared to 2015, and the majority of this population is expected to live in lower- and middle-income countries.

Ageing is the gradual decline of physical and mental capacity, and is a driver of various age-related diseases such as cardiovascular disease, cancer, immune system disorders, and musculoskeletal disorders [2]. Access to safe, nutritious and sufficient food is a fundamental human right for all people [3], and can contribute to improving long-term population, health, and national security. Secure access to food can produce wide-ranging positive impacts such as improved health and healthcare, poverty reduction, economic growth, job creation, and trade opportunities [4–6].

A longer, healthy life offers the promise of a healthy future for not only for older persons and their families, but also for societies as a whole. However, previous evidence suggests that food insecurity, particularly in older persons, still exists in many places of the world. In Portugal, 23% of older persons lived in a food-insecure household [7]. In the United States, food insecurity in older persons increased from 5.5% to 12.4% between 2007 and 2016 [8]. In Ethiopia, about 83% of households with older persons who receive pension beneficiaries experienced food insecurity [9, 10]. In South Africa, 30.2% of older persons lived with food insecurity [9–11]. In Tehran and Malaysia, 39.1% and 10.4% of older persons faced food insecurity, respectively [12, 13].

Various studies have shown that socio-economic and demographic characteristics of seniors are the main factors that support or threaten food security of the older population. Lack of food security or presence of food insecurity was more prevalent in lower-income, youngest-old age, less education, and unemployed groups [7, 8, 13]. There is also a significant relationship between financial difficulty and food security. Straining to make ends meet was associated with increased likelihood of an older person to be living in a food-insecure household [7]. Lobl et al. [14] found an association between having more financial stressors and increased risk of food insecurity in older persons in the United Kingdom, Germany, and the Netherlands. It has been also reported that the older persons with material hardship (e.g., debt) in the United States was linked to food insecurity [15].

Thailand is one of many ASEAN countries that has already become an “aged society” [16]. Almost 20% of total population were at age 60 years or older in 2021. This made Thailand the second most aged population in ASEAN after Singapore (22%) [17]. It is expected that in the next two decades the proportion of the Thai population that is older persons will exceed 31% of total population. The speed of ageing is projected to be highest in the oldest-old group (80+ years) [17]. Old-age dependency ratio (65+ per 15–59) of Thailand increased overtime, from 10.7 ratio in 1994 to 30.5 ratio in 2021 [18]. Consumption of healthy food is a crucial part of maintaining healthy behavior throughout life. Good nutrition will contribute to reduced risk of non-communicable diseases (NCD), better physiological wellbeing, and delayed care dependency [1]. However, unhealthy diets among Thai older persons have been observed. For example, nearly 70% of Thai older persons had insufficient intake of fruits and vegetables, and lived with chronic NCD and debilitating conditions [19].

The COVID-19 pandemic has been tremendous challenge for societies around the world, and has exposed the fragility of older persons’ health and livelihoods. Eleven percent of the total population infected with COVID-19, and 70% of COVID-19 deaths from among persons age 60 years or older [17]. In Thailand, the COVID-19 epidemic has disrupted food supply, and resulted in sudden loss of income and mounting debt among most households [20]. Thai household income declined significantly in the wake of COVID-19 [21]. The debt service rate increased from 29% in 2019 to 34% in 2021, and this is probably a direct cause of the decline in household consumption including food consumption in 2021. Over half (53%) of Thai older persons reported still working to earn income for feeding their family, paying debts, and supporting other household members [18]. This is presenting a major challenge for Thailand to meet the needs of an ageing society and, in particular, promotion of active and healthy ageing through financial resources and food security, for which older persons are always vulnerable.

As far as we know, there are no data in the literature regarding food security and financial hardship, or the association between these two in the ageing population in Thailand. Understanding the association between food security, financial hardship, and other socio-demographic factors is fundamental for improvement of geriatric health promotion policies and successful promotion of an active and healthy ageing population. Thus, the aim of this cross-sectional study was to investigate the association between food security, financial hardship, and other socio-demographic factors among older persons in Thailand.

## Methods

### Sampling and Study Design

This study used secondary data from a Cross-sectional Study on Fruit and Vegetable Eating Behaviors [22]. This nationally representative data was collected in 2021. The study selected a subsample of the dataset of people age 60 years or older for analysis. A multi-stage sampling design was conducted by the National Statistical Office to select the sample. The sampling process was undertaken sequentially across several hierarchical levels, first at the regional level, second at the provincial level, third at the district level, and last at enumeration area (EA). In total, nine provinces and one district were sampled. Within each district, EAs were randomly selected using a nationally-representative sampling frame of the national Population and Housing Census. Within each selected EA, 20 households were selected.

The survey included households in the four geographic regions of Thailand (central, north, northeast, and south) and Bangkok. In each region urban and rural localities were included. In total, 1,197 older persons were selected for this study.

### Data Collection

Data collection of the survey was done during June–December 2021 via face-to-face interviews using Qualtrics offline survey application. The survey was administered by trained research staff. Prior to the interview all respondents learned about the purpose of the study and overview of the survey process. Individual consent to participate was obtained before the interview started. The survey questionnaire included modules on socio-demographic characteristics, perceived financial hardship (debt burden, income problem, financial dissatisfaction), and food security.

### Outcome Variables

Individual food security was measured using the eight-question Food Insecurity Experience Scale (FIES), developed by the Food and Agriculture Organization (FAO) [23]. Eight questions (Supplementary Table S1) pertained to stages about individual’s experiences or behaviour related to insufficient resources to access food over the past 12 months. Each question refers to a different experience or behaviour, and thus a different level of severity of food insecurity—from mild to severe food insecurity. Food security is defined as 0 affirmative responses, meaning that an individual had sufficient resources for food at all times. Each respondent was asked to answer each question in order. If a respondent answered “no” for any question, then the respondent was instructed to immediately skip to the end of the FIES survey. The FIES score ranges from 0 (food secure) to 8 (severely food insecure). However, the purpose of this study was to measure prevalence of food security among older persons. Thus, researchers performed reverse scoring by re-coding the responses so that a high score is transformed into the corresponding low score on the scale. The reverse score therefore ranges from 0 (severely food insecure) to 8 (food secure).

This study used the validated Thai version of FIES by FAO and pretested it before actual data collection. Reliability of the Thai FIES questions were assessed using Cronbach’s alpha. The results for the assessment indicated good reliability of the questions, with alpha value of 0.89.

### Independent Variables

#### Socio-Demographic Characteristics

Sex was coded as male/female. Age was grouped as youngest-old (60–69 years), old-old (70–79 years), and oldest-old (80 years or over). Marital status includes married, single, or widowed/divorced/separated. Place of residence was classified into rural and urban areas. Regions of Thailand were divided into Central, North, Northeast and South. Educational attainment includes lower than primary school, primary school, secondary school, and bachelor’s degree or higher. Employment status was grouped as unemployed (people who are not in the labour force, jobless, actively seeking work, available to take a job), and employed (such as government employee, company-hired worker, business owner, wage labourer, and farmer).

#### Financial Hardship

The respondents were asked for their perception of their situation regarding the following financial constraints: debt burden, income problem, financial dissatisfaction. Debt burden was coded as “having no debt/having debt but not feeling burdened,” “having low debt burden” and “having high debt burden.” Income problem was coded as “no income problem,” “sometimes have an income problem” and “often have an income problem.” Financial dissatisfaction was coded as “dissatisfied,” “moderately satisfied” and “highly satisfied.”

### Statistical Analysis

This study first estimated the prevalence of food security among Thai older persons using descriptive statistics (i.e., frequencies and percentages). Next, the study examined differences in socio-demographic characteristics and financial hardship by an individual’s food security status using *t*-test. To examine the associations between an individual’s food security and other variables, multiple regression models were performed. All statistical tests were 2-sided, and statistical significance was considered at *p* < 0.05. Statistical analyses were performed using SPSS version 22.

Due to the fact that the economic factors in this study (i.e., employment status, debt burden, income problems, and financial dissatisfaction) may be highly correlated, the correlations between these variables were tested. The results in Supplementary Table S2 showed that their correlation coefficients were low (less than 0.7). Therefore, these variables were included in the final model.

## Results

### Prevalence of Food Security

The study found a mean score of food security of 7.0. Of total 1,197 older persons, 71% scored 8, meaning that they had food security. Almost one-third of the respondents had food insecurity and, of these, 1.3% had severe food insecurity (0 score). Table 1 shows the percentage of respondents by food security score from 0 to 8. Supplementary Table S3 provides an overview of characteristics of the sample by food security score.

**TABLE 1:** Food security score (*N* = 1,197) (Thailand, 2021).

Food security score	Severity scale	*N*	%	Mean score
0	Severe food insecurity 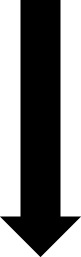 Food security	15	1.3	7.0
1		28	2.3	
2		42	3.5	
3		18	1.5	
4		9	0.7	
5		100	8.4	
6		34	2.9	
7		102	8.5	
8		850	71.0	

For persons who were experiencing food insecurity, the study found that 29% of respondents felt worried about not having enough food to eat, and 21% reported being unable to eat healthy and nutritious food in the past 12 months (Table 2). It is shown that 17.6% or slightly more than half of the respondents who experienced food insecurity ate only a few kinds of foods. Almost 9.3% of the respondents skipped their meal.

**TABLE 2:** Responses of older persons to each of the eight questions of the food insecurity experience scale (*N* = 1,197) (Thailand, 2021).

Question	*N*	%
Q1. You were worried you would not have enough food to eat?	347	29.0
Q2. You were unable to eat healthy and nutritious food?	245	20.5
Q3. You ate only a few kinds of foods?	211	17.6
Q4. You had to skip a meal?	111	9.3
Q5. You ate less than you thought you should?	102	8.5
Q6. Your household ran out of food?	84	7.1
Q7. You were hungry but did not eat?	43	3.6
Q8. You went without eating for a whole day?	15	1.3

Table 3 shows the distribution of the mean score of food security of respondents based on socio-demographic characteristics and financial hardship. Respondents who are male (7.21), at old-old age (7.26), single (7.15), and unemployed (7.05), live in urban area (7.07), and have bachelor’s or higher degree (7.83) had higher scores than other respondents in the same variable category. Respondents who reported having no debt (7.23), having no income problem (7.33) and feeling highly satisfied with their financial status (7.47) scored highest compared to other respondents in the same category.

**TABLE 3:** Characteristics of the sample and mean scores of their food security (Thailand, 2021).

Variables	N	%	Mean score	*t*-test(*p*-value)
Total	1,197	100.0	7.03	
Sex				***
Male	535	44.7	7.21	
Female	662	55.3	6.89	
Age group (years)				
60–69	716	59.8	6.93	
70–79	398	33.3	7.26	
80 or over	83	7.0	6.78	
Marital status				
Single	40	3.3	7.15	
Married	812	67.9	7.08	
Widowed/divorced/separated	345	28.8	6.91	
Place of residence				
Urban	498	41.6	7.07	
Rural	699	58.4	7.00	
Region				***
Bangkok	91	7.6	7.86	
Central	222	18.5	7.48	
North	374	31.3	6.75	
Northeast	225	18.8	6.32	
South	285	23.8	7.35	
Education attainment				***
Lower than primary school	199	16.6	6.55	
Primary school	872	72.9	7.06	
Secondary school	82	6.9	7.45	
Bachelor’s degree or higher	44	3.7	7.83	
Employment status				
Unemployed	688	57.5	7.05	
Employed	509	42.5	7.01	
Debt burden				***
No debt/having debt but not feeling burdened	860	71.8	7.23	
Low debt burden	101	8.5	6.95	
High debt burden	236	19.7	6.34	
Have an income problem				***
No problem	923	77.1	7.33	
Sometimes have an income problem	184	15.3	6.24	
Often have an income problem	90	7.5	5.57	
Financial dissatisfaction				***
Dissatisfied	285	23.8	6.08	
Moderately satisfied	559	46.7	7.24	
Highly satisfied	354	29.5	7.47	

Note(s): ***p ≤ 0.001.

There was an association between socio-demographic variables, in particular sex and education, and food security (*p* ≤ 0.001). All financial hardship-related variables had significant effects on the older persons’ food security status (*p* ≤ 0.001).

### Regression of Food Security on Financial Hardship and Socio-Demographic Factors

Table 4 shows results of the regression analysis that found statistically significant associations between all financial hardship variables, some socio-demographic characteristics and food security among the older persons.

**TABLE 4:** Multiple regression of food security score by financial hardship and socio-demographic characteristics (*N* = 1,197) (Thailand, 2021).

Variables	B	Std. Error	β	*p*-value
Sex (Reference group = Female)	0.282	0.112	0.073	*
Male				
Age group (Reference group = 80+ years)				
60–69 years	0.369	0.215	0.094	
70–79 years	0.607	0.214	0.149	**
Marital status (Reference group = Single)				
Married	0.210	0.293	0.051	
Widowed/divorced/separated	0.215	0.299	0.051	
Place of residence (Reference group = Urban)				
Rural	0.140	0.110	0.036	
Region (Reference = Bangkok)				
Central	−0.298	0.231	−0.060	
North	−0.841	0.222	−0.203	***
Northeast	−1.267	0.232	−0.257	***
South	−0.525	0.225	−0.116	*
Education (Reference group = Lower primary school)				
Primary school	0.504	0.141	0.117	***
Secondary school	0.616	0.235	0.081	**
Bachelor’s or higher degree	0.767	0.305	0.075	*
Work (Reference group = No)				
Yes	0.018	0.113	0.005	
Debt burden (Reference group = No debt/No burden)				
Low debt burden	−0.077	0.189	−0.011	
High debt burden	−0.264	0.143	−0.055	
Having income problem (Reference group = No problem)				
Sometimes having income problem	−0.730	0.152	−0.137	***
Often having income problem	−1.129	0.207	−0.155	***
Financial satisfaction (Reference group = Highly satisfied)				
Moderately satisfied	−0.093	0.122	−0.024	
Dissatisfied	−0.790	0.157	−0.175	***
Constant	6.920			
Adjusted R squared	0.174			

Note(s): *p ≤ 0.05, **p ≤ 0.01, ***p ≤ 0.001.

B, Unstandardized coefficient; β, Standardized beta coefficient.

The socio-demographic characteristics—sex, age, region, education—were found to relate to food security. Food security score was higher for being male, age 70–79 years and having at least primary education. Male respondents were more likely to have 0.282 (B = 0.282; *p* ≤ 0.05) greater food security scores than female. Respondents age 70–79 years had greater scores than those at age 80 years or over (B = 0.607; *p* ≤ 0.01). Respondents who live in the North, Northeast and South regions were more likely to have lower scores than those in Bangkok, especially people in the Northeast (B = −1.267; *p* ≤ 0.001). Respondents with at least primary school education had greater scores than those with less than primary school education. People who had at least a bachelor’s degree were more likely to have greater scores than other education groups (B = 0.767; *p* ≤ 0.05).

A statistically significant association of financial hardship was found with food security. Respondents reporting sometimes or often having an income problem were more likely to have lower scores than those with no income problem (B = −0.730; *p* ≤ 0.001 and B = −1.129; *p* ≤ 0.001, respectively). Financial dissatisfaction had statistically significant association with food security scores (B = −0.790; *p* ≤ 0.001, respectively).

## Discussion

Food security among older persons has become an important issue in improving health outcomes and geriatric care [7, 24]. The results of the present study show that, although many Thai older persons had food security, almost one-third of older persons still lived with food insecurity, and 1.3% experienced severe food insecurity. The study reported that food security was affected by various factors, in particular, financial vulnerability and certain socio-demographic characteristics, consistent with previous studies [7, 8, 24–27].

Food security in this sample was significantly higher among those who were male. This finding is consistent with several other reports that food security was more prevalent in male older persons than females [12–14, 27]. Traditionally, in Thai family culture, Thai men or fathers were expected to be leaders and providers of the family and were more involved in outdoor chores, whereas Thai women or mothers were expected to provide care and perform most of housework including procuring and preparing meals to promote overall family life and health, even at the expense of their own needs [28]. For example, the men in the traditional Thai household would eat first, followed by children and the adult women. Thus, even though women were expected to prepare the meals for the family, they were given lower priority for consumption.

Furthermore, this study found that food security tended to be more pronounced among older persons age 70–79 years than those at age 80 years or above. This may be explained by the impact of ageing which is “*a state of progressive functional decline accompanied by an increase in mortality* [29].” This state can lead to gradually functional decline in physical and mental capacities at greater age [30], and that affects the advanced older person’s ability to access to and eat various types of healthy and nutritious foods. This study also revealed that Thai older persons with low education tended to be the most vulnerable group for low food security. Previous studies found a significant association of the low levels of education (or the absence of formal education) of older persons with their cognitive performance, functional disability, and frailty [31]. This can limit their ability to manage food quantity and dietary quality which require more complex knowledge, skills, and behaviours for healthy eating, and thus become barriers to improved food security.

The lowest probability of having food security was found among older persons in the Northeast region. This was not surprising as the Northeast is the lowest with poverty line and highest with poor people among geographic regions in Thailand [32, 33]. This is consistent with previous studies that show that Thai people in the Northeast were most at risk of food security [34, 35]. This suggests special attention to the poorest and most vulnerable geographic region. The government needs to focus on small and specific strategies that work on improving food security, with full participation of key stakeholder in decision-making and policy development. This action is critical to reducing poverty in the region.

According the findings, income-related financial hardship were associated with food security among Thai older persons. These findings are comparable with studies conducted in other countries [14]. Financial hardship can push older persons further away from meeting standard living needs like buying food. This could limit their access to and impose financial constraints in consuming a healthy diet. Hardship would impact their ability to purchase enough food or nutritious food, which could result in risk of undernutrition and cardiometabolic disease if it occurs over a long period [36]. In addition, economic hardship or financial difficulties are important sources of financial stress which might increase the risk of experiencing depression [37]. Previous studies found that subjective financial measures are more direct indicators of financial stress than objective measures such as amount of debt or income [37]. This is because despite facing a similar objective financial situation, people may perceive or respond to their objective financial situation differently due to different experiences, personality and attitudes, resource management skills, and perceived financial-related sufficiency.

This study has some limitations. First, this was a cross-sectional study and, thus, causal inferences about the relationship between food security and other variables cannot be drawn. Future research which analyses the causal relationship between food security outcome and other factors over a period of time is needed. Second, the study focused on measuring food security rather than severity level of food insecurity, and that may limit a comprehensive understanding of food and diets of Thai older persons and thus limit for policy implications on the groups most vulnerable for low food security. Additional research specific to severity level of food insecurity is important in the future. Third, despite the significant relationship between food security and financial fragility, the effect of financial fragility on food insecurity in different socio-demographic characteristics of older persons (such as sex, age, marital status, place of residence, and education attainment) may differ. The study did not investigate the interactions between financial fragility and other independent variables. Therefore, further analysis is needed to test the interaction of these variables. Fourth, in addition to the analysis of debt and income related financial hardship in this study, other potential aspects should be analysed further, such as housing, health and family aspects that were found to be associated with food security in older age [14], and access to government’s welfare support to the older persons. This will help policymakers and other stakeholders to make decisions that are informed by a more comprehensive understanding and balanced view point in food security among financially fragile older persons.

### Policy Implications

This study has a number of implications for public policy around food security and financial circumstances among Thai older persons. *First*, the prevalence of food security is significantly lower in specific groups (e.g., female, oldest-old age, low level of education and Northeast residence). Government attention needs to be focused on development of specific actions or targeted interventions at older persons who are the most vulnerable groups for low food security, such as older women’s self-care and full participation in rural development and food security strategies, and social and community service provision addressing food security. It is also important for local governments to consult with different groups of older persons separately to understand their needs, and the degree to which they are able to access financial and non-financial resources, food and assistance. *Second*, the study highlights influence of income-related financial hardship on food security. This financial hardship could be used as a measure in improving food security and nutrition. In particular, the finding on the strong association of financial dissatisfaction and food security points to the importance of subjective financial measures in order to monitor and identify solutions for food security in older age. Other government’s support such as providing financial counselling services and financial education to those who are experiencing financial hardship should be implemented to help the fragile older persons manage with their financial problems including their concern and anxieties and associated food security more effectively. *Third*, factors contributing to food security are complex, interrelated, and operate beyond the individual level. Thus, improving food security among older persons requires cross-cutting strategies that all policy actors from various sectors need to address in their activities. This requires an interdisciplinary collaboration of policy actors from food, health, societal, and economic sectors and at the national and local level.

### Conclusion

This study found the association between perceived financial hardship, socio-demographic characteristics, and food security in the Thai ageing population. The results suggest that the presence of income-related financial hardship and anxieties affects low food security. The prevalence of food security in this population was also significantly associated with certain socio-demographic and geographic factors. These findings, in turn, could have important implications for policymakers and other stakeholders, especially the need for government and community support for those who are experiencing financial hardship and anxieties so that they can improve their economic situation and associated food security more effectively, such as financial counselling services and financial education. The government actions should also take into account the influence of socio-demographic and geographic factors among older persons, especially the most vulnerable groups for low food security. The findings also point out various factors beyond the individual level that contribute to older persons’ food security. Accordingly, there is a need for greater collaboration of policy actors across sectors such as health, food, societal, and economic sectors, that will be beneficial to developing targeted interventions either to alleviate financial hardship or improve food security among older persons.
