# Supplementary material for: Financial Hardship on Food Security in Ageing Populations
Source: Int J Public Health. 2023 Dec 14;68:1605755. doi: 10.3389/ijph.2023.1605755 (PMC10752981; doi:10.3389/ijph.2023.1605755)
Supplement: Supplementary file 1 [file Table1.docx]

**Table S1: Eight questions of the FIES for measuring severity of food insecurity**

(Source: Food and Agriculture Organization. The Food Insecurity Experience Scale Rome: Food and Agriculture Organization; n.d. [Available from: https://www.fao.org/in-action/voices-of-the-hungry/fies/en/].

| **Question** | Now I would like to ask you some questions about food. During the last 12 months, was there a time when: | | | | | | | |
| --- | --- | --- | --- | --- | --- | --- | --- | --- |
|  | Q1.  You were worried you would not have enough food to eat? | Q2.  You were unable to eat healthy and nutritious food? | Q3.  You ate only a few kinds of foods? | Q4.  You had to skip a meal? | Q5.  You ate less than you thought you should? | Q6.  Your household ran out of food? | Q7.  You were hungry but did not eat? | Q8.  You went without eating for a whole day? |
| **Label** | WORRIED | HEALTHY | FEWFOODS | SKIPPED | ATELESS | RANOUT | HUNGRY | WHOLEDAY |
| **Severity** | Mild **-------------------------------------------------->>------------------------------------------------>**  Severe | | | | | | | |
