# Supplementary material for: Financial Hardship on Food Security in Ageing Populations
Source: Int J Public Health. 2023 Dec 14;68:1605755. doi: 10.3389/ijph.2023.1605755 (PMC10752981; doi:10.3389/ijph.2023.1605755)
Supplement: Supplementary file 2 [file Table2.docx]

**Table S2: Correlation between Employment Status and Economic Hardship in Multiple Regression (N=1197)**

| **Variables** | | **Employment**  **Status** | **Debt**  **burden** | | **Have an income problem** | | **Financial dissatisfaction** | |
| --- | --- | --- | --- | --- | --- | --- | --- | --- |
|  |  |  | *Low debt burden* | *High debt burden* | *Sometimes have an income problem* | *Often have an income problem* | *Moderately satisfied* | *Highly satisfied* |
| **Employment**  **Status** | Pearson Correlation |  | .063* | .168** | .065* | .034 | .072* | .035 |
| **Low debt burden** | Pearson Correlation | .063* |  |  | .038 | .041 | .008 | -.011 |
| **High debt burden** | Pearson Correlation | .168** |  |  | .223** | .131** | -.043 | .279** |
| **Sometimes have an income problem** | Pearson Correlation | .065* | .038 | .223** |  |  | -.073* | .231** |
| **Often have an income problem** | Pearson Correlation | .034 | .041 | .131** |  |  | -.125** | .271** |
| **Moderately satisfied** | Pearson Correlation | .072* | .008 | -.043 | -.073* | -.125** |  |  |
| **Highly satisfied** | Pearson Correlation | .035 | -.011 | .279** | .231** | .271** |  |  |

*Correlation is significant at the 0.05 level (2-tailed).

**Correlation is significant at the 0.01 level (2-tailed).
