# Supplementary material for: Financial Hardship on Food Security in Ageing Populations
Source: Int J Public Health. 2023 Dec 14;68:1605755. doi: 10.3389/ijph.2023.1605755 (PMC10752981; doi:10.3389/ijph.2023.1605755)
Supplement: Supplementary file 3 [file Table3.docx]

**Table S3: Characteristics of the sample by food security score**

| **Variables** | **Food security score** | | | | | | | | |
| --- | --- | --- | --- | --- | --- | --- | --- | --- | --- |
|  | **0**  (N=15) | **1**  (N=28) | **2**  (N=42) | **3**  (N=18) | **4**  (N=9) | **5**  (N=100) | **6**  (N=34) | **7**  (N=102) | **8**  (N=850) |
| **Sex**  Male  Female | 27.8  72.2 | 36.6  63.4 | 29.0  71.0 | 44.2  55.8 | 27.7  72.3 | 44.0  56.0 | 39.1  60.9 | 36.4  63.6 | 47.5  52.5 |
| **Age groups**  60-69 years  70-79 years  80 years or over | 58.2  34.9  7.0 | 76.4  15.1  8.6 | 63.5  32.4  4.2 | 58.4  2.5  39.1 | 81.2  11.6  7.2 | 66.4  25.9  7.7 | 56.1  42.5  1.5 | 68.7  26.6  4.6 | 57.2  36.0  6.8 |
| **Marital status**  Single  Married  Widowed/divorced/separated | 0.0  66.6  33.4 | 2.4  67.0  30.6 | 4.2  68.6  27.2 | 7.7  52.9  39.4 | 0.0  64.8  35.2 | 2.2  60.7  37.2 | 1.1  69.1  29.8 | 4.4  68.2  27.4 | 3.4  69.0  27.6 |
| **Place of residence**  Urban  Rural | 40.0  60.0 | 45.7  54.3 | 55.0  45.0 | 63.7  36.3 | 90.6  9.4 | 39.6  60.4 | 47.1  52.9 | 26.4  73.6 | 43.6  56.4 |
| **Region**  Bangkok  Central  North  Northeast  South | 0.0  13.3  46.7  33.3  6.7 | 0.0  14.3  35.7  42.9  7.1 | 0.0  5.0  40.0  32.5  22.5 | 0.0  11.1  55.6  11.1  22.2 | 0.0  11.1  0.0  44.4  44.4 | 1.0  9.9  44.6  30.7  13.9 | 2.9  5.7  40.0  40.0  11.4 | 2.0  9.8  34.3  22.5  31.4 | 10.1  22.3  28.0  14.1  25.4 |
| **Education attainment**  Lower than primary school  Primary school  Secondary school  Bachelor’s or higher degree | 50.8  47.3  1.9  0.0 | 26.2  69.8  4.1  0.0 | 19.9  76.0  3.2  0.9 | 10.0  78.0  12.0  0.0 | 6.4  93.6  0.0  0.0 | 26.7  70.6  2.3  0.4 | 24.3  65.8  8.7  1.1 | 18.2  74.9  3.6  3.2 | 14.1  73.2  8.0  4.6 |
| **Employment status**  Unemployed  Employed | 55.5  44.4 | 56.0  44.0 | 68.0  32.0 | 60.8  39.2 | 52.9  47.1 | 43.2  56.8 | 39.6  60.4 | 35.2  64.8 | 42.3  57.7 |
| **Debt burden**  No debt / No burden  Low debt burden  High debt burden | 35.7  11.1  53.3 | 59.0  13.0  28.0 | 51.7  12.5  35.8 | 71.1  2.8  26.1 | 44.3  0.0  55.7 | 63.4  8.5  28.1 | 61.7  6.0  32.3 | 61.6  4.5  33.8 | 76.8  8.8  14.4 |
| **Having income problem**  No problem  Sometimes having income problem  Often having income problem | 45.8  32.2  22.1 | 41.8  30.2  28.1 | 49.5  30.4  20.2 | 69.4  20.7  9.8 | 79.0  11.4  9.6 | 52.9  29.6  17.4 | 67.6  15.6  16.8 | 60.9  27.8  11.2 | 85.5  10.6  3.9 |
| **Financial satisfaction**  Dissatisfied  Moderately satisfied  Highly satisfied | 63.9  20.8  15.4 | 54.8  37.6  7.6 | 54.1  31.3  14.7 | 30.6  31.9  37.5 | 25.2  52.1  22.7 | 40.8  43.5  15.7 | 39.7  46.5  13.8 | 41.5  38.5  20.0 | 15.7  49.8  34.5 |
